# A potentiated startle study of uncertainty and contextual anxiety in adolescents diagnosed with autism spectrum disorder

**DOI:** 10.1186/2040-2392-4-31

**Published:** 2013-09-04

**Authors:** Paul D Chamberlain, Jacqui Rodgers, Michael J Crowley, Sarah E White, Mark H Freeston, Mikle South

**Affiliations:** 1 Neuroscience Center, Brigham Young University, S192 ESC, Provo, UT 84602, USA; 2 Clinical Psychology, Institute of Neuroscience, Ridley Building, Newcastle University, Newcastle NE1 7RU, UK; 3 Yale Child Study Center, Yale University School of Medicine, 230 South Frontage Road, New Haven, CT 06520, USA; 4 Center for Neuroscience, University of California-Davis, 1544 Newton Court, Davis, CA 95618, USA; 5 Department of Psychology, Brigham Young University, 245 TLRB, Provo, UT 84602, USA

**Keywords:** Autism spectrum disorder, Anxiety, Fear, Potentiated startle, Eye blink, EMG, Intolerance of uncertainty, Psychophysiology, Repetitive behavior

## Abstract

**Background:**

Beyond the core symptoms of autism spectrum disorder (ASD), associated symptoms of anxiety can cause substantial impairment for individuals affected by ASD and those who care for them.

**Methods:**

We utilized a potentiated startle paradigm with a puff of air to the neck as the unconditioned stimulus in order to investigate differences between response to cued fear and contextual anxiety among cognitively able adolescents diagnosed with ASD and an age- and IQ-matched typically developing group.

**Results:**

In a threat-modulated startle paradigm, response patterns to neutral, predictable, and unpredictable conditions were comparable across typically developing and ASD youth in terms of startle response magnitude and latency. However, the ASD group showed significantly greater absolute startle responsivity at baseline and throughout the experiment, suggesting possibly enhanced general sensitivity to threatening contexts. The ASD group, but not the control group, demonstrated moderate to strong negative correlations between psychophysiological response to unpredictable threats (uncertainty) and questionnaire measures of generalized anxiety, intolerance of uncertainty, and repetitive behavior.

**Conclusions:**

Our data suggest enhanced general reactivity among the ASD group, possibly reflecting greater sensitivity to the threatening context of the startle paradigm. Associations with the response to uncertainty may help explain shared neurobehavioral mechanisms in ASD and anxiety. This task can provide useful targets for future neuroimaging and genetics studies as well as specific avenues for intervention. We emphasize the importance of further basic and clinical research into links among these important constructs.

## Background

Autism spectrum disorder (ASD) refers to a collection of neurodevelopmental conditions characterized by impaired social communication and repetitive, stereotyped patterns of behavior [1]. In addition to these core symptoms, there is a high prevalence of anxiety concerns encompassing all diagnostic subtypes of anxiety [2,3]. Anxiety-related impairment may often be as significant as, or greater than, the difficulties arising from the autism symptoms *per se*[4]. Clarifying the mechanisms that link anxiety with the core symptoms of autism is important for basic etiological research and may also refine targets for behavioral and pharmacological treatment [5].

### Relationship of anxiety to core symptoms of autism

Several recent lines of work have explored the relationship between core symptoms of repetitive behavior in ASD and associated anxiety-related constructs. Rodgers and colleagues [6] found evidence for a relationship between parent reports of elevated anxiety in ASD children and increased repetitive behaviors, especially with ‘insistence on sameness’ in routines and interests. Rodgers and colleagueshypothesize that the association between anxiety and repetitive behavior in ASD may be mediated at least in part by an ‘intolerance of uncertainty’ that arises due to atypical information processing, including basic sensory processing, that is common in ASD. Intolerance of uncertainty (IU) underlies a perceptual bias associated with beliefs about worry that become more potent in ambiguous contexts [7]. A substantial literature implicates IU in the maintenance of generalized anxiety disorder (GAD). IU may arise from atypical information processing, including basic sensory processing, common to ASD. This atypical information processing in ASD impairs integration of environmental cues, leading to increased perception of ambiguity in ASD across multiple contexts. This in turn leads to increased worry and subsequent anxiety, including generalized anxiety.

Relatedly, two recent studies reported atypical responding in reversal learning paradigms. South *et al*. [8] paired one of two simple cues (colored squares) with a puff of air delivered to the neck in a sample of 30 older children and adolescents diagnosed with ASD and an age- and IQ-matched typically developing youth. Both groups demonstrated comparable acquisition of a classical fear conditioning measured through differential skin conductance responding to threat versus safety cues. However, the ASD group was significantly delayed in reacquiring fear to the reversed threat and safety cues. Moreover, significant correlations emerged between psychophyiological measures of reversal learning - when relatively simple rules for learning an experimental task are switched without warning - and questionnaire-based reports of repetitive behavior in ASD samples. These associations were unique to repetitive behaviors and were not found for other symptom domains. Similarly, D’Cruz *et al*. [9] reported behavioral results from a reward-based probabilistic learning task among 41 ASD participants including children and adults with matched controls. Participants chose a stimulus based on location with 80% of correct choices and 20% of incorrect choices randomly reinforced. After making the correct choice over multiple trials, the rewarded stimulus location changed without warning (reversal). D’Cruz *et al*. also observed intact contingency acquisition of the task in ASD but reported that, while the ASD participants tended to initially make correct post-switch choices, they ‘regressed’ to previous choices more often than controls.

Together these studies suggest that straightforward learning of initial rules or contexts is often intact, at least in high-functioning ASD samples, but that learning may break down under more complex situations, those with increased ambiguity and uncertainty. A recent review of the literature by Gaigg [10] argues that atypical emotion processing early in life in ASD disrupts a range of cognitive processes, including social cognition. As noted above, both the South *et al*. [8] and D’Cruz *et al*. [9] studies observed associations specifically with measures of everyday behavioral rigidity. Nonetheless it is possible these may also reflect indirect effects on social function, because social interactions are inherently dynamic. Attenuated social perceptual skills in ASD [11] may accentuate the unpredictability and aversiveness of social situations. For the present study, we utilized a paradigm that was created explicitly for the study of uncertainty in other clinical populations [12], in order to directly test the psychophysiological consequences of contextual uncertainty in ASD.

### Differentiating fear and anxiety

Psychophysiological studies devoted to understanding the underlying mechanisms for anxiety in ASD began with a number of classical fear conditioning studies using either skin conductance response [4,13] or fear-potentiated startle [14-16] as dependent measures. However, a growing body of translational work supports the distinction between ‘cued fear’ as assessed in the studies noted above, and ‘sustained fear’ that is associated with anxiety disorders [17]. More specifically, fear is a state of apprehension that begins with the presentation of a potentially threatening stimulus and then subsists in the absence of such a stimulus. Anxiety (also called ‘contextual fear’ or ‘contextual anxiety’) is characterized by apprehension that is present even in the absence of a threatening stimulus.

The neural substrates for fear and anxiety are thought to be dissociable. An important review by Davis and colleagues [18] suggested that the amygdala is involved in stimulus-specific fear, while activity in the bed nucleus of the stria terminalis (BNST; sometimes called the ‘extended amygdala’) is associated with anxiety. Animal work has shown that disruption of the central nucleus of the amygdala (the main amygdalar output) prevented the expression of cued fear-potentiated startle (FPS). However, similar studies performed on the BNST resulted in no such potentiation to a cued stimulus. By contrast, light-enhanced startle (indicative of sustained anxiety) can be disrupted in the BNST, but not in the central nucleus of the amygdala. Studies in humans implicate the amygdala for initial acquisition of the fear response and orbitofrontal cortex for updating information about fear context; disruption in amygdala-orbitofrontal cortex feedback systems may underlie sustained anxiety [19].

Schmitz and Grillon [12] have recently provided a detailed protocol for using neutral, predictable and unpredictable aversive events to study behavioral manifestations of fear *vis-á-vis* anxiety in humans. The NPU (neutral, predictable, unpredictable)-threat task (NPUTT) exposes participants to three different contexts: 1) neutral condition: a cue appears, but never an aversive stimulus; 2) predictable condition: an aversive stimulus (an air puff for children; shock for adults) is explicitly predicted in this environment by a cue; 3) unpredictable condition: an aversive stimulus is administered with no connection to the appearance of a cue.

A startling white noise probe is administered to the participant both during the presentation of each cue and during the intertrial interval (ITI; that is, between presentations of the cues). The startle response is measured by the eyeblink reflex using electromyography (EMG) sensors. Startle response magnitude during the ITI is considered to be a measure of contextual fear/anxiety, and is expected to increase linearly between the neutral, predictable, and unpredictable environments, respectively. That is, contextual anxiety increases as the amount of uncertainty present in the task increases. This task is distinctly different from the conditioning tasks used in previous ASD research [14,16], which utilize cued fear rather than contextual anxiety to potentiate the startle reflex. The NPUTT and similar tasks have been used by Grillon and colleagues in a variety of studies involving anxious patient populations. These studies have shown for example that individuals with posttraumatic stress disorder (PTSD) generalize fear across stimuli [20]; children of anxious patients have abnormal startle reactivity [21], women have greater sustained anxiety but not phasic fear than men [22], and contextual anxiety but not cued fear is elevated in clinical anxiety [23].

To our knowledge, no studies to date have applied the NPUTT or a similar uncertainty paradigm to an ASD population. Given the potential relevance of uncertainty to symptoms of anxiety in ASD, we compared NPUTT response across a sample of older adolescents diagnosed with ASD and age- and ability-matched controls. We hypothesized that children with ASD are less able to adapt to contextual threat and uncertainty than typically developing children and therefore would show increased startle response compared to controls during the intertrial intervals (when the probe can happen any time) during both the predictable and unpredictable phases; and relatively greater magnitude of response during the unpredictable versus predictable phase, reflecting overall greater anxiety during times of uncertainty. We also hypothesized that measures of everyday behavioral rigidity and intolerance of uncertainty would be more strongly and positively associated with startle response to uncertainty in the ASD group than the control group.

## Methods

### Participants

Our initial sample included 49 older adolescents between the ages of 15 to 18. Ten participants (five ASD) were excluded from further analysis due to unresponsiveness to the startle probe, defined as exhibiting a startle response in less than 50% of trials. The final sample included 18 participants diagnosed with an autism spectrum disorder (the ASD group; 17 male) and 21 typically developing participants (the CON group; 15 male). Diagnosis of an ASD was determined by an expert diagnostician using DSM-IV criteria, based on information obtained from the Autism Diagnostic Observation Schedule (ADOS) Module 3 or 4 and from the parent-report Social Responsiveness Scale (SRS). An IQ estimate was determined using the Wechsler Abbreviated Scale of Intelligence. All participants above the age of 18 and the parents of those under age 18 gave written informed consent as approved by the Brigham Young University Institutional Review Board. All participants were compensated for their time.

### Associated variables

Participants completed several measures of anxiety and rigidity via a digital survey. These included the Spence Children’s Anxiety Scale-child report (SCAS-C; [24], a reliable and valid measure of child anxiety that reports both an overall total score and six subscale scores). We were most interested in the GAD subscale, which includes items such as ‘I worry about things’ and ‘When I have a problem, I feel shaky’. We used a shortened, 12-item version of the Intolerance of Uncertainty Scale (IUS) [S. Walker, ‘What do we know about the relationship between intolerance of uncertainty and worry in young children?’, unpublished PhD thesis] including questions such as ‘When things happen very suddenly, I get upset’ and ‘Feeling unsure stops me from doing most things’. One parent of each participant completed a parent-report version of the same measures (that is SCAS-P, IUS-P). We also examined a key measure of behavioral rigidity from the SRS, the Autistic Mannerisms subscale (for example, ‘when under stress, he or she shows rigid or inflexible patterns of behavior that seem odd’ and ‘has more difficulty than other children with changes in his or her routine’). In order to test specificity of these hypothesized associations, we also tested general associations with social disability (the SRS Total Score), IQ, and participant age.

### Stimuli and apparatus

Stimulation was controlled by E-prime 2.0 Professional Software (Psychology Software and Tools, Inc., Sharpsburg, PA, USA). The acoustic startle stimulus was a 40-ms duration, 103 dB burst of white noise presented binaurally through headphones. Participants wore a vest that closed with Velcro straps and had a ½ inch (~2.54 centimeter) firm-yet-flexible hose threaded up through the vest and adjusted to point toward the junction of the neck and the chin, with a gap of about 8 centimeters between the end of the tube and the skin. The approximately 3-meter- long tube was connected to a tank of compressed ‘room’ air with a medical-grade valve and regulator set to deliver a puff of air to the neck at 75 psi (measured at the regulator valve). E-prime software controlled an electronic switch that delivered each puff for 100 ms. Prior to the start of the experiment, participants received two puffs of air to familiarize them with the stimulus and give them the chance to opt out if they wanted. Participants were also reminded they could stop the experiment at anytime if they chose to.

The startle reflex was measured using two 8 millimeter silver-silver chloride electrodes placed beneath the left eye, over the obicularis oculi muscle, one centered beneath the pupil and a second positioned immediately lateral to that toward the outer canthus. A third, grounding electrode was placed on the left forearm. Prior to placement of the electrodes, the skin was cleaned and then abraded using NuPrep skin gel (Weaver and Company, Aurora, CO, USA). Electrodes were attached to a BIOPAC MP150 EMG100C module (BIOPAC Systems, Inc., Goleta, CA, USA), and data was recorded on a Macintosh MacBook Pro laptop (Apple Inc., Cupertino, CA, USA) using AcqKnowledge 4.0 software (BIOPAC Systems, Inc.). The electromyogram was acquired with amplifier bandwidth set at 28 to 500 Hz with a sampling rate of 2,000 Hz.

### Experimental design

**Figure 1:**
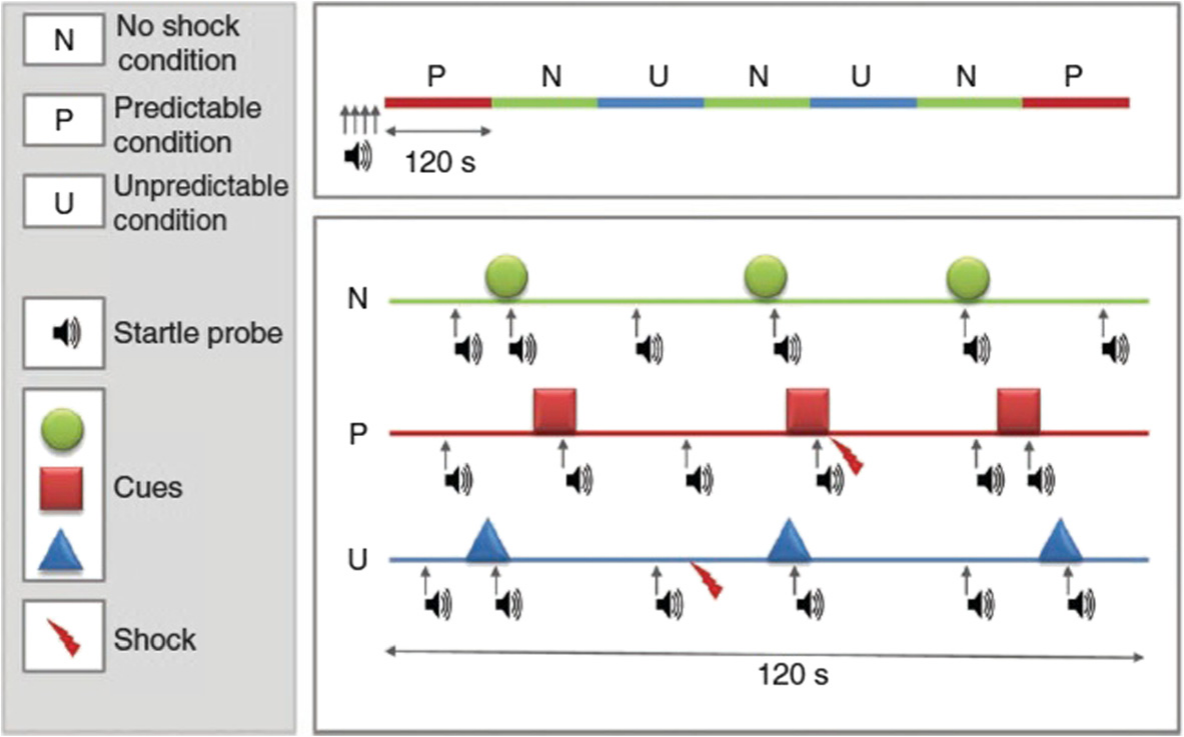
**Schematic representation of sequences of stimulus presentation during each condition in one block of the NPU-threat test.** The upper part of the figure represents a complete block including two P (predictable), two U (unpredictable) and three N (no shock) conditions. The lower part shows examples of each condition, including startle probes, cues (8 s duration) and shocks. Used by permission from [12].

The experimental design followed the protocol published by Schmitz and Grillon [12] in *Nature Protocols*. Testing consisted of a beginning habituation phase of nine startle probes (103 dB, 40 ms duration), followed by two recording blocks separated by a 5-minute break. Each block consisted of four initial habituating startle probes followed by three neutral (N), two predictable (P), and two unpredictable (U) conditions. These conditions were presented in one of the following orders: P N U N U N P or U N P N P N U. Block order was randomized across participants with 9 CON (of 21) and 8 ASD (of 18) participants receiving the block beginning with the P condition first. A diagram of the protocol is presented in Figure 1.

Each condition (that is N, P, and U) lasted approximately 120 s during which time a cue (green circle for N condition; red square for P condition; blue triangle for U condition) was presented three times for 8 s each time. When the cue was absent, a white fixation cross was present on the screen the entire time to help maintain the participant’s attention. In addition to the cue or fixation cross, the top of the monitor displayed a reminder of the condition for the participant. These read ‘No puff’, ‘Puff only during red square’, and ‘Puff at any time’, for the N, P, and U conditions, respectively. Because this was not a conditioning experiment, the reminder was necessary to ensure that differences in response to the startle probe in each condition were not due to differences in learning across participants.

Startle probes were administered six times during each 120 s condition: during each of the three cues, and during each ITI. Startle probes were separated by at least 20 s. Air puffs were administered once or twice per P (0.5 s before the end of the cue) and U condition (pseudo-randomized during the absence of the cue) resulting in six air puffs per block and twelve air puffs throughout the course of the experiment. Schmitz and Grillon [12] have shown previously that absence of a shock during the cue in the U condition does not lead to safety learning.

Between blocks, participants had a 5-minute break to rest. During this time participants were asked to report their anxiety level retrospectively during each of the three conditions, with and without the cue present. Ratings were reported using Qualtrics online survey software (Qualtrics, Provo, UT, USA). Each question displayed the image associated with that condition (P, U, or N) either with or without the cue, and included a text-based numbered rating scale from 1 to 10, with 1 labeled as ‘not anxious/fearful’ and 10 labeled as ‘extremely anxious/fearful’. The same survey was repeated following the completion of the second block.

### Data analysis

Data extraction was performed using Acqknowledge software and BIOPAC’s Eyeblink Startle Scoring application notes (application note number AS214). Raw EMG data were subjected to a 60 Hz band stop filter and a copy of the EMG signal was integrated for further processing. Peak amplitude of the blink reflex was determined in the 27 to 150 ms window following startle probe onset. Response magnitudes were transformed into *t* scores and then averaged within condition (for example, ITI P, cue P, ITI U, cue U, ITI N, cue N). The latency-to-peak amplitude for each window was collected and analyzed in the same manner.

*T* scores for EMG magnitude during the cue conditions were entered into a 2 (diagnosis) × 3 (threat phase) ANOVA. Previous research on the cue phases (see [12]) predicted a main effect for condition with greater startle in the P condition compared to the N and U conditions. Because of previous research noted above that showed normal potentiated startle in ASD in response to a specific threatening stimulus, we did not predict a diagnosis x condition interaction in the presence of the cue.

Contextual threat was analyzed using the ITI data for EMG magnitude in a similar 2 × 3 ANOVA. Here we predicted a main effect for condition with greater startle in the U condition compared to the N and P conditions, as has been found in other studies of the NPUTT [12]. We also expected those with ASD to respond more intensely to uncertainty than controls, and predicted a diagnosis x condition interaction.

*T* scores or other standardization procedures are useful for controlling for the contributions of raw baseline variability, and are standard procedure for answering the question of how different phases of uncertainty affect relative response within each person [12]. However, in light of variable findings in previous studies of psychophysiological reactivity in ASD, we believed it would also be interesting to examine the raw EMG scores, with the addition of baseline reactivity as another measurement phase. Raw scores for the latency-to-peak amplitude of each blink response were likewise analyzed in 2 × 3 ANOVAs for cued and ITI conditions.

To test hypothesized associations among our primary outcome variables of interest (the cued, predictable condition and the uncued, unpredictable condition), we planned correlation analyses between these variables and measures of anxiety, uncertainty, and rigid repetitive behavior along with potential confounds of social dysfunction, IQ, and age. We planned follow-up regression analyses for resulting variables with the best likelihood of predicting outcome.

## Results

### Baseline measures

As shown in Table 1, there were no significant between-group differences in either chronological age or Full Scale IQ. As expected, the ASD group scored significantly higher on parent-report measures of autism symptoms, anxiety, and IU. There were no significant differences in participant questionnaire reports of air-puff-related anxiety or intensity following either block of the task (all *P*s >0.25).

**Table 1:** Participant characteristics

	**M**		**SD**		**Range**		***t***
**Measure**	**ASD**	**CON**	**ASD**	**CON**	**ASD**	**CON**	
Age (years)	16.64	16.99	0.98	0.96	15-18	15-18	1.09
Full-scale IQ	104.82	108.74	13.32	10.56	85-124	92-129	0.98
SRS total	103	17.87	23.65	10.70	68-150	2-38	12.86^***^
SRS mannerisms	19.06	2.88	5.91	3.10	9-29	0-11	9.76^***^
ADOS total	13.11	--	3.80	--	7-18	--	--
SCAS-P total	24.78	10.28	14.54	8.53	3-58	2-18	4.37^***^
SCAS-C total	26.28	22.22	10.54	10.16	2-53	6-40	1.18
IUS-P	40.47	22.47	11.28	7.79	24-58	12-40	5.18^***^
IUS-C	33.94	30.22	8.39	7.45	20-48	18-44	1.41

M, Mean; SD, Standard Deviation; ASD, autism spectrum disorder; CON, typical control; Full-scale IQ, from the Wechsler Abbreviated Scales of Intelligence; SRS, Social Responsiveness Scale; ADOS, Autism Diagnostic Observation Schedule (Module 3 or 4); SCAS-P/-C, Spence Children’s Anxiety Scale parent report/child report); IUS-P/-C, Intolerance of Uncertainty Scale parent report/child report). ***P <.001.

### Psychophysiological response

**Figure 2:**
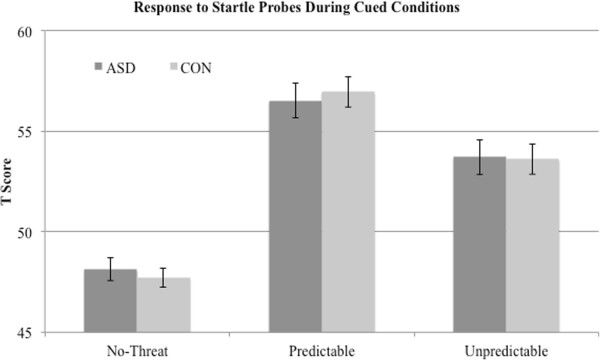
EMG magnitude to startle probes during cued phases shows expected main effect for condition type (P >U >N) with a similar between-group response.

**Figure 3:**
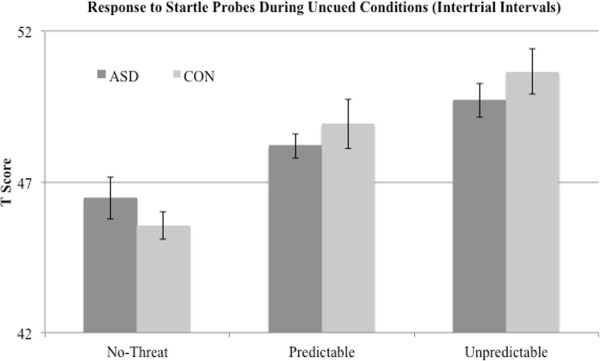
EMG magnitude to startle probes during the uncued intertrial intervals shows the expected main effect for condition type (U >P >N) with a similar between-group response.

*T* scores of blink magnitude during the cue phases showed the expected main effect of condition, (F(2,70) = 62.03, *P* <0.001, η^2^ = 0.64). The *a priori* prediction (see [13]) of P >U >N was confirmed and the linear contrast was significant (F(1,37) = 120.983, *P* <0.001, η^2^ =0.77). As can be seen in Figure 2, responses were identical for both groups following the 2 × 3 ANOVA. There was no main effect for group (F(1,35) = 0.00, *P* = 0.96, η^2^ = 0.00) or group x condition interaction (F(2,70) = 0.18, *P* = 0.83, η^2^ = 0.01). A similar 2 × 3 ANVOA for the ITI (uncued) phases likewise demonstrated a significant main effect for condition (F(2,70) = 15.45, *P* <0.001, η^2^ = 0.31). As noted above, for the ITI phase it is expected that the contextual anxiety will be highest in the between-cue times, when the timing of the air puff is uncertain, U >P >N. This expected pattern was confirmed (Figure 3) and the linear contrast was significant (F(1,37) = 32.61, *P* <0.001, η^2^ = 0.47). There was again no main effect for group (F(1,35) = 0.41, *P* = 0.53, η^2^ = 0.01) or group x condition interaction (F(2,70) = 0.84, *P* = 0.44, η^2^ = 0.02). Because of the imbalance in the ratio of males to females across groups we reran these analyses for main effects and interactions with only males in each group; results were identical in showing significant main condition effects but no group x phase interactions for both cued and ITI conditions.

**Figure 4:**
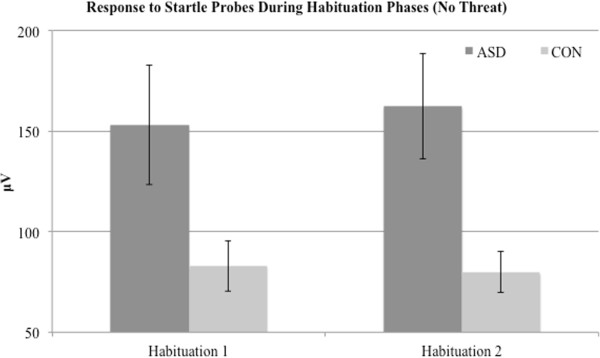
Absolute startle magnitude during habituation phases shows the ASD group is significantly more reactive to the white noise probes outside of the task threat context.

Next, we performed the same analyses using raw blink magnitude instead of *t* scores. The relative pattern of responses for EMG magnitude across the N, P, and U phases was identical for the *t* score analyses of both cued and ITI conditions. However, there was an additional, significant main effect for diagnostic group in both cued and ITI phases. Interestingly, the ASD group showed overall increased magnitude relative to controls: cued condition F(1,35) = 5.50, *P* <0.05, η^2^ = 0.14; ITI F(1,35) = 4.59, *P* <0.05, η^2^ = 0.12. In order to ascertain whether this group difference was due to differing task-related response or was present more generally, we analyzed both baseline and post-break time habituation phases: as shown in Figure 4, the ASD group showed substantially increased responses during both habituation phases (baseline *t*(35) = 2.32, *P* <0.05; post-break *t*(35) = 2.92, *P* <0.01).

Given these differences in baseline, we redid the analyses of raw blink magnitude using response to the first habituation phase as a covariate (suggested by [13] as an alternate to using standardized *t* scores). No results changed in this analysis. For the cued phase, the main effect held (F(2,70) = 3.29, *P* <0.05, η^2^ = 0.09) with no main effect for group (F(1,35) = 0.64, *P* = 0.43, η^2^ = 0.02) or group x condition interaction (F(2,70) = 0.20, *P* = 0.82, η^2^ = 0.01). This was likewise true for the ITI phase: main effect (F(2,70) = 5.09, *P* <0.01, η^2^ = 0.13) with no main effect for group (F(1,35) = 0.25, *P* = 0.62, η^2^ = 0.01) or group x condition interaction (F(2,70) = 0.12, *P* = 0.89, η^2^ = 0.00).

### Latency

Results for the analyses of latency-to-peak showed the identical pattern as findings from the cued magnitude scores: condition (F(2,70) = 6.87, *P* <0.01, η^2^ = 0.16), P >U >N linear contrast (F(1,37) = 16.17, *P* <0.001, η^2^ = 0.30); group effect (F(1,35) = 0.03, *P* = 0.86, η^2^ = 0.00); group x condition interaction (F(2,70) = 1.28, *P* = 0.28, η^2^ = 0.04). In the ITI condition, latency-to-peak analyses showed no significant effects: condition (F(2,70) = 1.41, *P* = 0.25, η^2^ = 0.04, group (F(1,35) = 1.10, *P* = 0.30, η^2^ = 0.03); group x condition interaction (F(2,70) = 0.37, *P* = 0.68, η^2^ = 0.01).

### Relationship with associated variables

We first ran correlation analyses with our primary cued fear and contextual anxiety outcome variables, using the magnitude *t* scores. There were no reliable correlations between our outcome measures and overall level of social disability (SRS Total Score), IQ, or parent-reported IU. For the ASD group only, there were significant or near-significant (*P* <0.06) associations for the anxiety condition (uncued, unpredictable phase) for the remaining variables (listed in order of the value of the correlation coefficients): SCAS-C total anxiety (r = -0.59, *P* = 0.011), SCAS-C GAD (r = -0.57, *P* = 0.015), SCAS-P GAD scores (r = -0.46, *P* = 0.052), SRS Mannerisms (r = -0.45, *P* = 0.059) and the IU child report (r = -0.45, *P* = 0.059). Participant age neared a significant correlation with the cued, predictable outcome variable (r = 0.46, *P* = 0.052). We then conducted a step-wise regression analysis for these variables, in the order just listed, to test predictive power for our startle response outcomes.

None of these variables significantly predicted the response to fear (cued, predictable condition) for either diagnostic group. However, a model including all six of these variables significantly predicted the anxiety condition (uncued, unpredictable) for the ASD group only, F (6,11) = 3.53, *P* = 0.034. Several of the included variables did not add significantly to the predictive value of that model. A final model consisting of SCAS-C GAD (generalized anxiety), SRS Mannerisms (rigid, repetitive behavior) and IUS-C (intolerance of uncertainty) scores had the strongest predictive power for startle response during the uncertainty condition, F (3,14) = 6.45, *P* = 0.003. This model is shown in Table 2 for both the ASD and CON groups.

**Table 2:** Linear regression model with dependent variable as the EMG startle response to the unpredictable condition during the uncued intertrial interval, considered to be the context marked by the highest level of uncertainty

**Measure**	**B**	**SE B**		**β**	**R**^**2 **^**change**	
ASD model						Total adjusted R^2^ = 0.53, *F* (3,14) = 6.45, *P* = 0.003
SCAS-C GAD	-0.39	0.18		-0.39	0.32^*^	
SRS Mannerisms	-0.19	0.07		-0.45	0.13^§^	
IUS-C	-0.12	0.05		-0.43	0.17^§^	
CON model						Total adjusted R^2^ = -0.14, *F* (3,13) = 0.35, *P* = 0.79
SCAS-C GAD	-0.57	0.60	-0.36	0.03		
SRS mannerisms	-0.22	0.32	-0.20	0.03		
IUS-C	0.08	0.16	0.17	0.02		

^*^P <0.05, ^§^*P* <0.10. ASD, autism spectrum disorder; SCAS-C GAD, generalized anxiety disorder on the Spence Children’s Anxiety Scale-child report; SRS Mannerisms, parent report rigid and repetitive behavior on the Social Responsiveness Scale; IUS-C, Intolerance of Uncertainty Scale-child report; CON, typical control.

## Discussion

Recent work on potential mechanisms contributing to high rates of anxiety in autism spectrum disorder (ASD) underscores the relevance of an intolerance of uncertainty (IU). IU has long been implicated in studies of clinically anxious patients, especially those struggling with generalized anxiety, not affected by autism [7,24]. We utilized a sensitive, fear-potentiated startle (FPS) paradigm to investigate psychophysiological response to induced uncertainty in an experimental paradigm. Contrary to our hypotheses, adolescents diagnosed with ASD showed comparable responsivity to a matched control group for predictable threat, unpredictable threat, and no impending threat conditions, for both EMG magnitude and latency. While the NPUTT differs from conditioning tasks because there is no learning of association involved and because of the explicit demarcation of predictable and unpredictable threat, these results add to others [14,16] to suggest that FPS is intact in ASD. Because startle responsivity during the intertrial interval of the NPUTT (representative of contextual threat) was comparable to controls, we may infer that its neural substrates, particularly the BNST, function normally for this type of assessment. Recently South *et al*. [8] observed delayed reversal learning despite intact fear conditioning in ASD. They proposed that anxiety symptoms in ASD may be related more to feedback mechanisms (for example, from the orbitofrontal cortex) rather than bottom-up processes (for example, amygdala activity). While our findings do not necessarily support or contradict the possibility of orbitofrontal cortex contributions to anxiety in ASD anxiety, they do provide additional support for the conclusion that ASD anxiety is not amygdala, or extended amygdala (BNST) based.

Despite similar startle response patterns across ASD and controls, specific patterns related to perceptions of uncertainty may involve atypical thresholds and/or pathways in ASD. First, we were surprised to find that the ASD group was significantly more reactive than controls, in terms of raw startle amplitude, during baseline and all phases of the experiment. Although a very early study of 10 ASD individuals and 10 controls did find increased heart rate and skin conductance response to environmental stimuli [25], previous skin conductance work on fear conditioning has found no difference in baseline skin conductance response in ASD vs. controls [see [4,8]. It may be that FPS measurements reflecting reflexive responding to an abrupt sensory event may be more sensitive than SCR for detecting the threshold to potentially threatening environmental stimuli and/or contexts.

Gaigg and Bowler [13] suggest another possible interpretation, that inconsistencies across previous studies concerning psychophysiological responses to concrete stimuli may be explained in terms of the ambiguity of such stimuli, in that unambiguous stimuli elicit typical arousal responses while ambiguous ones elicit variable responses in ASD. The group differences in this study, however, were found in the habituation phases where there was no direct threat context. Could there be a generalized uncertainty created by the task, and that uncertainty about when a stimulus will be followed by an air puff creates more ambiguity? That is, the ASD group exhibits heightened baseline levels of arousal because the experimental protocol on the whole induces a level of uncertainty, because the different conditions are essentially randomized in the entire session. This raises an interesting prediction, namely that individuals with ASD can cope more easily with short-term uncertainties but not longer-term uncertainties (such as those found in the social environment). Future research designs may explicitly test Gaigg and Bowler’s hypothesis using FPS as a dependent variable.

Grillon *et al*. [26], see also [27] have suggested that unpredictability only contributes to emotional reactivity for stimuli that are sufficiently aversive. Whereas Sterling *et al*. [16] delivered their air puff at 60 psi, ours was at 75 psi^a^, and the extra strength of our air puff may have crossed that level uniquely for the ASD group. Because Sterling *et al*. did not report on their response to the Habituation phase, direct comparison of this possibility during baseline conditions is not feasible. Clearly, further research is needed on reactivity in ASD samples both before and during experimental manipulations. Attention to baseline contexts and to methodological choices - including reporting of both raw and standardized data, which highlight distinct important aspects of task response - will be important for elucidating whether there may be a subtle but important psychophysiological hypersensitivity in ASD.

This study arises directly from our previous study of reversal learning following classical fear conditioning. We likewise referenced the D’Cruz *et al*. study [9] of probabilistic reversal. While the reversal component is an important similarity, the paradigms are sufficiently different that we may not predict similar results in a startle paradigm. In the D’Cruz task, participants are required to make a choice on every trial. Thus each trial induces uncertainty whether that choice will lead to positive or negative reinforcement. In contrast, in our startle paradigm, the participants are passive viewers and never make a choice. Also, outcomes are always aversive; the uncertainty pertains to the timing of the aversive event, not whether it will occur. That uncertainty did not affect the ASD group in our study as it did in the D’Cruz study may be due to these key differences in decision-making parameters.

There is a large gap between everyday behavior (reported through questionnaires) and results from laboratory-based experimental studies [17,28]. When reliable associations are found it is not always clear what they mean. As noted in our introduction, multiple lines of evidence have led us to focus on the interplay of anxiety, repetitive behavior, and IU in ASD, but the core processes that may link these together have not been adequately studied. The associations we report here suggest that these pathways are important for further study. The negative relationships between EMG and measures of IU, repetitive behavior and GAD symptoms were against prediction. However, in a recent study, typically developing adolescents who were selected as either relatively high or low on IU completed the Iowa Gambling Task while rating subjective anxiety at each block and while SCR was recorded [A Wild, MH Freeston, S Heary & J Rodgers, ‘Diminished physiological flexibility is associated with intolerance of uncertainty during affective decision making in adolescence*,’* under review]. The authors report that those who were high in IU reported higher subjective anxiety but lower SCR response relative to the low group. These findings are reminiscent of studies with adults with GAD who, despite showing increased baseline arousal compared to controls, show less responsiveness to laboratory stress [29] and on an ambulatory monitoring task [30], suggesting decreased physiological flexibility.

Paradigms arising from analog animal studies are important for elucidating the neural basis for many aspects of behavior in ASD, including anxiety and repetitive behavior [31]. This task and similar ideas lend themselves well to functional neuroimaging (for example, using EEG and/or functional magnetic resonance imaging (fMRI)) and we plan to utilize these techniques in the near future. On the other hand, there is an urgent need for studies with greater ecological validity [28]. Recent studies of social stressors such as public speaking [32,33] mark an important step forward. There is a need for ecologically valid studies of nonsocial stressors that might capture more of the generalized anxiety and other forms of worry (for example, perfectionism) frequently seen in ASD. Understanding where ASD is similar to and different from typical development is also critical for reward as well as fear pathways [34-36].

To date, FPS studies in autism have included only small samples such as this one. While it is more difficult to undertake studies using FPS than with simpler measures such as skin conductance, larger samples will be needed to more thoroughly model response to fear, anxiety, and related variables. A strength of our study was a relatively narrow age range, compared to most previous studies in the area. Nonetheless, our older (15 to 18 years), cognitively able ASD sample (IQ mean = 104) may have developed compensatory strategies for underlying ASD-related differences in response to fear and anxiety-provoking situations. Younger samples will shed more light on the developmental trajectory of anxiety in ASD and more impaired samples may elucidate where autism-related behaviors are more salient than anxiety-related problems. We also recommend studies that explicitly manipulate the potency of the anxiety-provoking stimulus. The similarity between ASD and CON groups in this study may simply be a consequence of the task working so well that almost all individuals responded to the task, and more subtle thresholds of difference in ASD were overwhelmed by the potency of the task (including the strength of the air puff itself and the manipulation of cued and uncued/unpredictable puffs). Whereas most people will respond to clear danger signals, it is likely that part of the anxiety response in ASD is related to environmental signals that most people do not find threatening. Clever within-subject designs are needed investigate this possibility. The greater ratio of females in our control group may also be a limitation, especially given literature showing more anxiety in females relative to males, although analyses conducted with only males showed similar results.

## Conclusions

For many individuals and families affected by autism spectrum disorder, frequent and pervasive worry and anxiety cause substantial distress [2,4]. Although similar brain systems (especially the amygdala) have been implicated in both social impairment and symptoms of anxiety in ASD, a number of studies are showing that, at the least, the acquisition of a fear response is intact in ASD (but see [13] for an important exception). Therefore high rates of anxiety are not likely related to atypical recognition (either hyperarousal or hypoarousal) of threatening stimuli. Alternative possibilities include difficulties with feedback mechanisms, for instance in orbitofrontal cortex or difficulties monitoring or integrating information in circuits involving, for example, anterior cingulate cortex and insula cortex [8,37,38]. Rodgers and colleagues have proposed a model relating sensory behavior, repetitive behavior, and anxiety in ASD to the ‘intolerance of uncertainty’ concept that has been important for conceptualizing non-autism clinically anxious groups. In conjunction with studies arising directly from animal models (for example, [9]) and paradigms that focus on ecological validity [28], studies that explicitly investigate the mechanisms of underlying anxiety in ASD promise to improve understanding of autism etiology as well as to specify avenues for improved treatment of anxiety-related distress.

## Endnote

^a^Studies using air puffs commonly vary in intensity between 60 and 100 psi. The puff used in our study was in no way painful but was quite startling.

## Abbreviations

ADOS: Autism diagnostic observation schedule; ASD: Autism spectrum disorder; BNST: Bed nucleus of the stria terminalis; EMG: Electromyography; FPS: Fear-potentiated startle; GAD: Generalized anxiety disorder; ITI: Intertrial interval; IU: Intolerance of uncertainty; IUS-C/-P: Intolerance of uncertainty scale child report/parent report); NPU: Neutral, predictable, unpredictable; NPUTT: Neutral, predictable, unpredictable-threat task; SCAS-C/-P: Spence children’s anxiety scale child report/parent report); SRS: Social responsiveness scale.

## Competing interests

The authors declare no competing financial interests or other potential conflicts of interests.

## Authors’ contributions

PDC and MS conceived of the study, programmed the task and oversaw data collection and extraction, assisted with data analysis, and drafted the manuscript. JR and MHF initiated discussion of intolerance of uncertainty in autism spectrum disorder, contributed to study design and analysis, and reviewed the manuscript. MJC provided valuable expertise on EMG data collection and analysis as well as manuscript review. SEW directed our theoretical discussions of uncertainty into measureable constructs, and provided programming assistance as well as drafting of the manuscript. All authors reviewed and approved the manuscript.
